# Integrated Analysis of Long Non-coding RNAs (lncRNAs) and mRNAs Reveals the Regulatory Role of lncRNAs Associated With Salt Resistance in *Camellia sinensis*

**DOI:** 10.3389/fpls.2020.00218

**Published:** 2020-03-19

**Authors:** Siqing Wan, Yongheng Zhang, Mengsha Duan, Linli Huang, Weidong Wang, Qingshan Xu, Yajun Yang, Youben Yu

**Affiliations:** ^1^College of Horticulture, Northwest A&F University, Yangling, China; ^2^Tea Research Institute, Chinese Academy of Agricultural Sciences, National Center for Tea Improvement, Key Laboratory of Tea Biology and Resources Utilization, Ministry of Agriculture, Hangzhou, China

**Keywords:** *Camellia sinensis*, long non-coding RNA, salt stress, antisense oligodeoxynucleotide suppression, endogenous target mimic

## Abstract

Tea plant (*Camellia sinensis*), an important economic crop, is seriously affected by various abiotic stresses, including salt stress, which severely diminishes its widespread planting. However, little is known about the roles of long non-coding RNAs (lncRNAs) in transcriptional regulation under salt stress. In this study, high-throughput sequencing of tea shoots under salt-stress and control conditions was performed. Through sequencing analysis, 16,452 unique lncRNAs were identified, including 172 differentially expressed lncRNAs (DE-lncRNAs). The results of Gene Ontology (GO) and Kyoto Encyclopedia of Genes and Genomes (KEGG) enrichment analyses of their cis- and trans-target genes showed that these DE-lncRNAs play important roles in many pathways such as the galactinol synthase (GOLS), calcium signaling pathway, and interact with transcription factors (TFs) under salt stress. The data from the gene-specific antisense oligodeoxynucleotide-mediated reduction in the lncRNA *MSTRG.139242.1* and its predicted interacting gene, *TEA027212.1* (*Ca^2+^-ATPase 13*), in tea leaves revealed that *MSTRG.139242.1* may function in the response of tea plants to high salinity. In addition, 12 lncRNAs were predicted to be target mimics of 17 known mature miRNAs, such as *miR156*, that are related to the salt-stress response in *C. sinensis*. Our results provide new insights into lncRNAs as ubiquitous regulators in response to salt stress in tea plants.

## Introduction

Tea plant [*Camellia sinensis* (L.) O. Kuntze] is an important economic crop in many countries, such as China, Japan, and India. Tea made from tea plant leaves is one of the most consumed drinks worldwide. However, tea plants are seriously affected by many abiotic stresses, such as cold, salt, and drought stresses, due to their specific environmental requirements, which severely diminish the widespread planting of tea plants (Upadhyaya and Panda, 2013). Therefore, it is important for the tea industry to breed tea plant species that exhibit strong resistance and adaptability. Salt stress is a form of abiotic stress that severely affects the growth of tea plants and the quality of tea products (Upadhyaya and Panda, 2013). In our previous report, we performed an Illumina RNA sequencing (RNA-Seq) to compare the transcriptomes of tea plants treated with and without NaCl, and many differentially expressed mRNAs involved in signal transduction pathways, transcription factors (TFs), and other functional genes under salt stress were identified (Wan et al., 2018). Previous reports have also shown that many mRNAs and proteins in tea plants, such as *CsSnRK2* (Zhang et al., 2018), *CsAQP* (Yue et al., 2014), and *CsVQ* (Guo et al., 2018), respond to salt stress. Long non-coding RNAs (lncRNAs) are important regulatory factors that respond to various abiotic stresses in plants; however, their functions in responding to salt stress have not been studied in tea plants. Therefore, the identification of important lncRNAs that respond to salt stress in tea plants is necessary.

LncRNAs are non-coding RNAs that are longer than 200 nt and usually have low protein-coding potential (Mercer et al., 2009). According to their genomic origins, lncRNAs are broadly divided into four types: intronic lncRNAs, intergenic lncRNAs (lincRNAs), sense lncRNAs, and antisense lncRNAs (Liu et al., 2012). LncRNAs are considered “transcriptional noise” because of their poor conservation and the limited evidence for their functions (Ponjavic et al., 2007). However, increasing research has revealed that lncRNAs function in transcriptional regulation and epigenetic gene regulation, and some lncRNAs function as cis- or trans-regulators in various biological processes (Ponting et al., 2009). In mammals, many lncRNAs have been proven to be related to a wide range of diseases, especially cancers and neurodegenerative diseases (Ma et al., 2012). In recent years, a large number of lncRNAs that are important in plant growth and development (Heo and Sung, 2011; Ding et al., 2012), biotic stress responses (Cui et al., 2017), and abiotic stress responses (Swiezewski et al., 2009; Qin et al., 2017) have been identified. For example, the reduced expression of the rice lncRNA *LDMAR* can lead to male sterility under long-day conditions in rice (Ding et al., 2012). In *Arabidopsis*, the antisense lncRNA *FLORE* can regulate the circadian clock to photoperiodic flowering (Henriques et al., 2017). A nucleus-localized lncRNA, *DRIR*, can enhance drought- and salt-stress tolerance in *Arabidopsis* (Qin et al., 2017). Currently, with the development of deep-sequencing technology, genome-wide lncRNA analyses have been performed on many species, such as *Arabidopsis* (Liu et al., 2012), rice (Zhang Y.C. et al., 2014), *Zea mays* (Boerner and McGinnis, 2012), wheat (Shumayla et al., 2017), and other species (Zhang and Chen, 2013). However, in many non-model plants, studies on lncRNAs are rather limited. Recently, Varshney et al. (2019) discovered 33,400 putative lncRNAs in different tissues of tea plants, which was the first report of lncRNAs in tea plants.

In this study, to explore the early response of lncRNA under salt stress in tea plants, we reanalyzed the transcriptomes of tea plants treated with and without NaCl based on the latest tea tree genome (Wei et al., 2018). LncRNAs were identified and classified systematically. The functions of these lncRNAs were predicted, and differentially expressed lncRNAs (DE-lncRNAs) that responded to salt stress were identified. In addition, the cis-targeting and trans-targeting relationship between lncRNAs and mRNAs and the possible interactions between lncRNAs and abiotic stress-related miRNAs were found to be involved in salt stress in tea plants. Notably, in our previous report, the Ca^2+^-transporting ATPase was shown to be involved in the salt-stress response in tea plants (Wan et al., 2018), and in this study, the differentially expressed Ca^2+^-transporting ATPase 13 (*TEA027212.1*) was proven to be co-expressed with the lncRNA *MSTRG.139242.1*, which indicates that this lncRNA may participate in Ca^2+^ signal transduction in response to salt stress. In addition, six DE-lncRNAs were randomly selected for quantitative real-time PCR (qRT-PCR) validation to confirm the reliability of the expression levels obtained from the RNA-Seq transcriptome. Overall, the results obtained in our study provide a valuable resource for studying lncRNAs involved in salt stress in tea plants and will enhance our understanding of the putative regulatory functions of lncRNAs in plants.

## Materials and Methods

### Plant Materials and NaCl Treatments

One-year-old “Pingyangtezao” tea plant cutting seedlings with consistent growth were pre-incubated in nutrient solution under standard growth conditions (temperature: 25 ± 3°C, air relative humidity: 60–70%, photoperiod: 12 h light/12 h dark) for 2 months. These tea plants were used for stress assays under the same growth conditions. In order to analyze the early response of tea plant to high salinity and evaluate the early salt responsive genes in the tea plant leaves, salt treatment was conducted by soaking the roots in nutrient solution that contained 250 mM NaCl (salt treatment) or standard nutrient solution (control), and fresh leaf samples (first and second leaves) were randomly collected at 4 h when tea plants began to show the symptoms of salt stress and wilt slightly after treatment. Control and NaCl treatment were both repeated three times (Control1, Control2, and Control3 and Salt1, Salt2, and Salt3). The samples were immediately immersed in liquid nitrogen and stored at −80°C until RNA-Seq, qRT-PCR validation, and further analysis.

### Analysis of Transcriptomic Data Based on the Tea Tree Reference Genome

**FIGURE 1:**
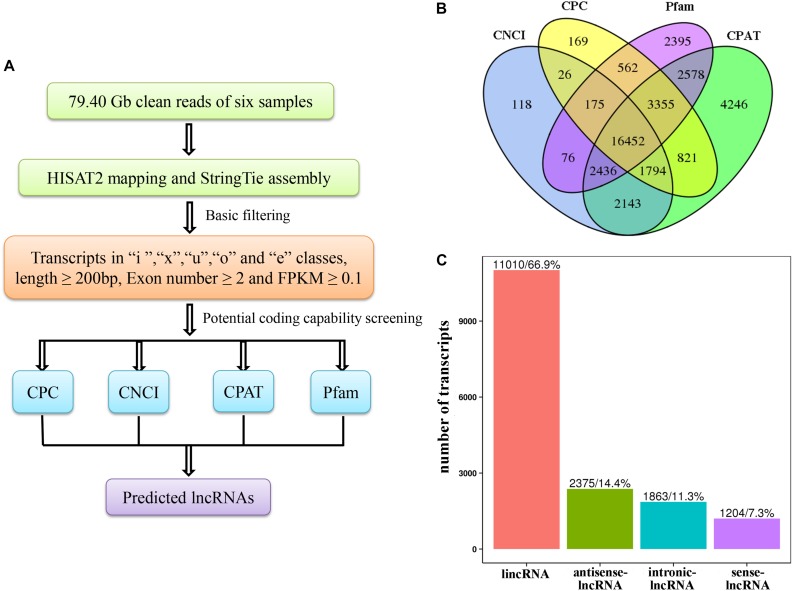
**(A)** Pipeline for the identification of lncRNAs in *Camellia sinensis*. **(B)** Filtering of the candidate long non-coding RNAs (lncRNAs). Venn diagrams of coding potential analysis using four methods [Coding Potential Calculator (CPC), Coding-Non-Coding Index (CNCI), Coding Potential Assessment Tool (CPAT), and Protein family (Pfam)]. Those simultaneously shared by the four methods were predicted as candidate lncRNAs and used in subsequent analyses. **(C)** Classification of lncRNAs.

In our previous report, we sequenced six tea plant samples using RNA-Seq technology. A total of 79.40 Gb of clean data was obtained, and the Q30 base percentage of each sample was greater than 92.93% (Wan et al., 2018). The identification of lncRNAs was executed according to the pipeline shown in Figure 1A. All RNA-Seq datasets were aligned to the reference genome of *C. sinensis* var. *sinensis* (Wei et al., 2018) using the HISAT2 system^1^ (Kim et al., 2015) to reconstruct the transcriptome. After the alignment, the StringTie software^2^ (Pertea et al., 2016) was used to assemble reads into transcripts and for quantification. The assembled transcripts were annotated using the gffcompare program,^3^ and the unknown transcripts were used to screen for putative lncRNAs.

### Identification of LncRNAs in Tea Plants

The prediction of lncRNAs consisted of two parts: basic filtering and potential coding capability screening. For basic filtering, transcripts with a class code of “i,” “x,” “u,” “o,” and “e” (Lv et al., 2013), a length ≥ 200 bp, an exon number ≥ 2, and a fragments per kilobase of transcript per million mapped reads (FPKM) value ≥ 0.1 (Kelley and Rinn, 2012) were selected. Subsequently, four methods, including Coding Potential Calculator (CPC)^4^ (Kong et al., 2007), Coding-Non-Coding Index (CNCI)^5^ (Sun L. et al., 2013), Coding Potential Assessment Tool (CPAT)^6^ (Wang et al., 2013), and Protein family (Pfam)^7^ (Finn et al., 2014) protein domain analysis were used to evaluate the coding capabilities of the transcripts, and potential lncRNAs were filtered out by combining the four results. Next, the identified lncRNAs were classified into four categories: lincRNA, antisense-lncRNA, intronic-lncRNA, and sense-lncRNA using cuffcompare.^8^ In addition, lncRNAs and mRNAs were comparatively analyzed according to their transcript length and exon number.

### Prediction of Potential Target Genes of LncRNAs

The prediction of potential target genes of lncRNAs was based on the two interaction modes between lncRNAs and mRNAs. First, because lncRNA can regulate the expression of nearby genes, we searched coding genes 100 kb upstream and downstream of lncRNAs for cis-target genes. The other interaction mode of lncRNA and mRNA is due to the complementary pairing of bases. The LncTar (Li et al., 2015) tool was used for target gene prediction of lncRNAs. The free energy and standard free energy of paired sites were calculated, and the target genes with standard free energy threshold < −0.1 were considered trans-target genes of lncRNAs.

### Identification and Functional Analysis of DE-LncRNAs

The expression levels of lncRNAs were quantified using FPKM values with the StringTie software. Differential expression analysis of the NaCl and Control groups was performed using the DESeq R package (1.10.1).^9^ The resulting *P*-values were adjusted using Benjamini and Hochberg’s approach for controlling the false discovery rate. LncRNAs with an adjusted value of *P* < 0.01 and an absolute value of log2 (fold change) > 1 found by DESeq were considered as differentially expressed.

Functional analyses of DE-lncRNAs were performed by annotation and classification of their cis- and trans-target genes. BLASTX alignment (value of E < 10^–5^) (Altschul et al., 1997) between target genes and public databases was performed, which included the Clusters of Orthologous Groups (COG) of proteins (Tatusov et al., 2000) and Kyoto Encyclopedia of Genes and Genomes (KEGG) databases (Kanehisa et al., 2004). Gene Ontology (GO) classifications were conducted using Blast2GO and WEGO software (Ashburner et al., 2000).

### Prediction of LncRNAs as Endogenous Target Mimics for miRNAs Under Salt Stress

The 172 DE-lncRNAs and 539 known conserved mature miRNAs of *C. sinensis* (Zhu and Luo, 2013; Zhang Y. et al., 2014; Jeyaraj et al., 2017a, b; Sun et al., 2018) were submitted to psRNATarget^10^ using a maximum expectation of 2.0 (Wu et al., 2013; Dai et al., 2018) for miRNA–lncRNA interaction prediction. Less than three mismatches and G/U pairs were allowed within the lncRNA and miRNA pairing regions. The co-expression network was established using Cytoscape,^11^ based on the DE-lncRNAs, target mRNAs of DE-lncRNAs, and miRNAs interacting with DE-lncRNAs.

### LncRNA and Target mRNA Suppression in Tea Plants Using Antisense Oligonucleotides (AsODNs)

A gene-suppression assay in tea plants using antisense oligonucleotides (AsODNs) was conducted according to Liu et al. (2018). ODN sequences were selected using the Soligo software.^12^ The sequences of input and selected ODNs are shown in Supplementary Data Sheet S1: Table S1. To increase the efficiency of gene suppression, two or three independent ODNs were synthesized of each gene and mixed in equal proportions during treatment. The ODNs were purified by HPLC, and the concentration was diluted to 10 μM. Fresh tea shoots with a bud and two leaves were excised and inserted into centrifuge tubes containing 1 ml of mixed ODNs for treatment. All leaves from the shoots were harvested after 24 h of treatment, and each treatment had three biological repeats. At the same time, a random non-sense control was used (Supplementary Data Sheet S1: Table S1). A nucleotide BLAST search of the tea tree genome CDS and lncRNA sequences with this sequence was used to verify that the sequences did not overlap with other sequences.

### qRT-PCR

qRT-PCR was performed to validate the expression patterns of salt-responsive lncRNAs. Total RNA was isolated from the six tea samples (Salt1, Salt2, Salt3, Control1, Control2, and Control3) using TRNzol reagent (TIANGEN, Beijing, China). First-strand cDNA was synthesized using the PrimeScript RT reagent kit with gDNA Eraser (TaKaRa, Dalian, China), after which qRT-PCR detection was completed using the EvaGreen qPCR MasterMix-No Dye kit (ABM, Richmond, BC, Canada) on a StepOne Plus PCR instrument (ABI, United States) following the manufacturers’ protocols. The tea plant *Cs*β*-actin* gene was chosen as the reference gene in accordance with previous methods (Wan et al., 2018), and the primers used in this assay are shown in Supplementary Data sheet S1: Table S2. The relative expression levels of the genes were calculated using the 2^–ΔΔ*CT*^ method (Livak and Schmittgen, 2001), and the data are presented as the mean ± SD from three independent biological replicates. Group differences were tested using one-way ANOVA and Duncan’s test, and significant differences among various treatment groups are represented by different letters (*P* < 0.05). The SPSS 20.0 software was used to determine significant differences between the Control and 250 mM NaCl treatment data.

## Results

### Identification of LncRNAs in *C. sinensis*

To identify salt-responsive RNAs in *C. sinensis*, six cDNA libraries from three controls (Control1, Control2, Control3) and three salt-treated (Salt1, Salt2, Salt3) leaf samples that were treated with 250 mM NaCl for 4 h were constructed and sequenced using an Illumina HiSeq2500 platform in our previous report (Wan et al., 2018). In this report, the RNA-Seq results were aligned to the latest tea tree genome, and the alignment results are shown in Table 1. The mapped ratio of each sample ranged from 88.99 to 91.10%. After the data were filtered with CPC, CNCI, Pfam, and CPAT, 16,452 candidate lncRNAs were obtained from the six samples (Figure 1B). Then, the identified lncRNAs were classified into four categories: 11,010 lincRNAs (66.9%), 2,375 antisense-lncRNAs (14.4%), 1,863 intronic-lncRNAs (11.3%), and 1,204 sense-lncRNAs (7.3%) (Figure 1C).

**TABLE 1:** Overview of the genome alignment result.

Sample	Total reads	Mapped reads	Uniq mapped reads	Multiple mapped reads	Reads map to “ + ”	Reads map to “−”
Control1	112,129,142	101,328,235 (90.37%)	70,079,537 (62.50%)	31,248,698 (27.87%)	3,8880,996 (34.68%)	3,8760,991 (34.57%)
Control2	130,302,020	118,699,443 (91.10%)	76,309,916 (58.56%)	42,389,527 (32.53%)	43,511,636 (33.39%)	43,518,412 (33.40%)
Control3	78,517,558	71,255,097 (90.75%)	47,395,231 (60.36%)	23,859,866 (30.39%)	26,782,952 (34.11%)	26,888,454 (34.25%)
Salt1	119,801,158	106,611,134 (88.99%)	80,043,176 (66.81%)	26,567,958 (22.18%)	43,845,279 (36.60%)	43,746,111 (36.52%)
Salt2	84,340,792	75,281,525 (89.26%)	54,731,132 (64.89%)	20,550,393 (24.37%)	30,080,568 (35.67%)	29,993,645 (35.56%)
Salt3	110,082,884	98,490,021 (89.47%)	71,042,037 (64.54%)	27,447,984 (24.93%)	39,127,799 (35.54%)	39,106,854 (35.52%)

**FIGURE 2:**
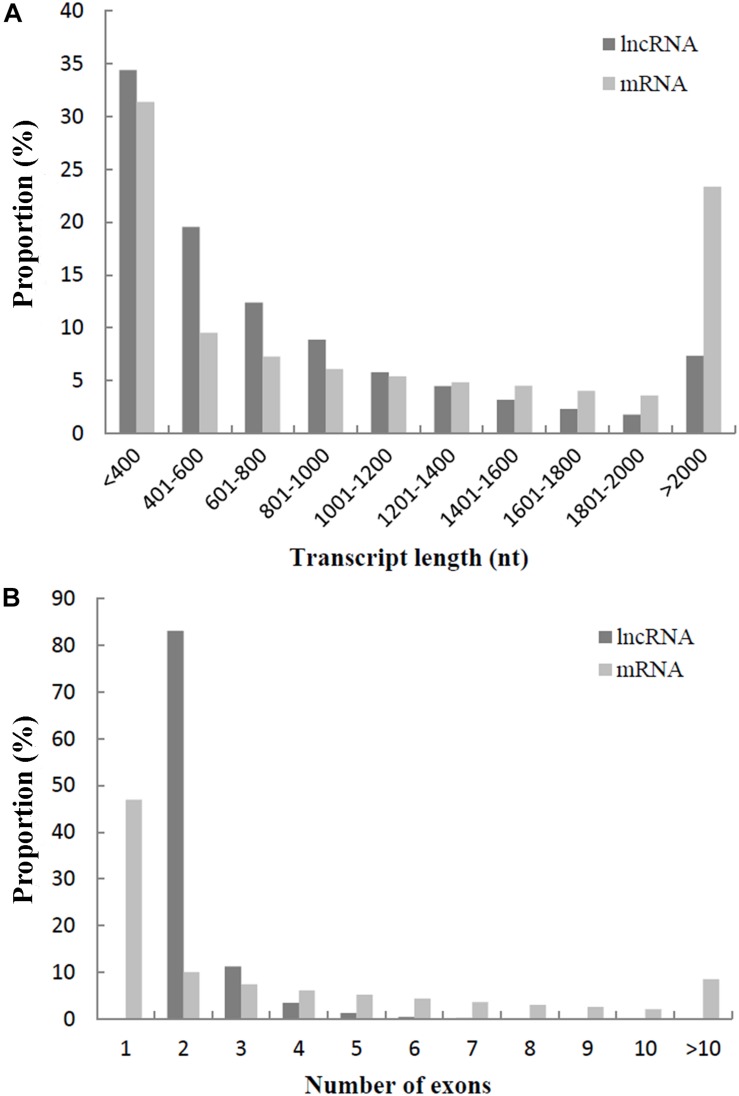
**(A)** Comparison of transcript length between lncRNAs and mRNAs. **(B)** Comparison of exon number between lncRNAs and mRNAs.

To understand the differences between lncRNAs and mRNAs in sequence and structure, lncRNAs were analyzed and compared with mRNAs, according to the transcript length and number of exons. The average length of lncRNAs was 822 nucleotides, and the average length of mRNAs was 1,348 nucleotides. As shown in Figure 2A, 75.19% of lncRNAs were < 1,000 nucleotides, while only 54.28% of mRNAs were < 1,000 nucleotides. In addition, 94.44% of lncRNAs had two (83.15%) or three (11.29%) exons, while 10.05% of mRNAs had two exons and 46.99% of mRNAs had only one exon (Figure 2B).

### DE-lncRNAs Involved in NaCl Stress

**FIGURE 3:**
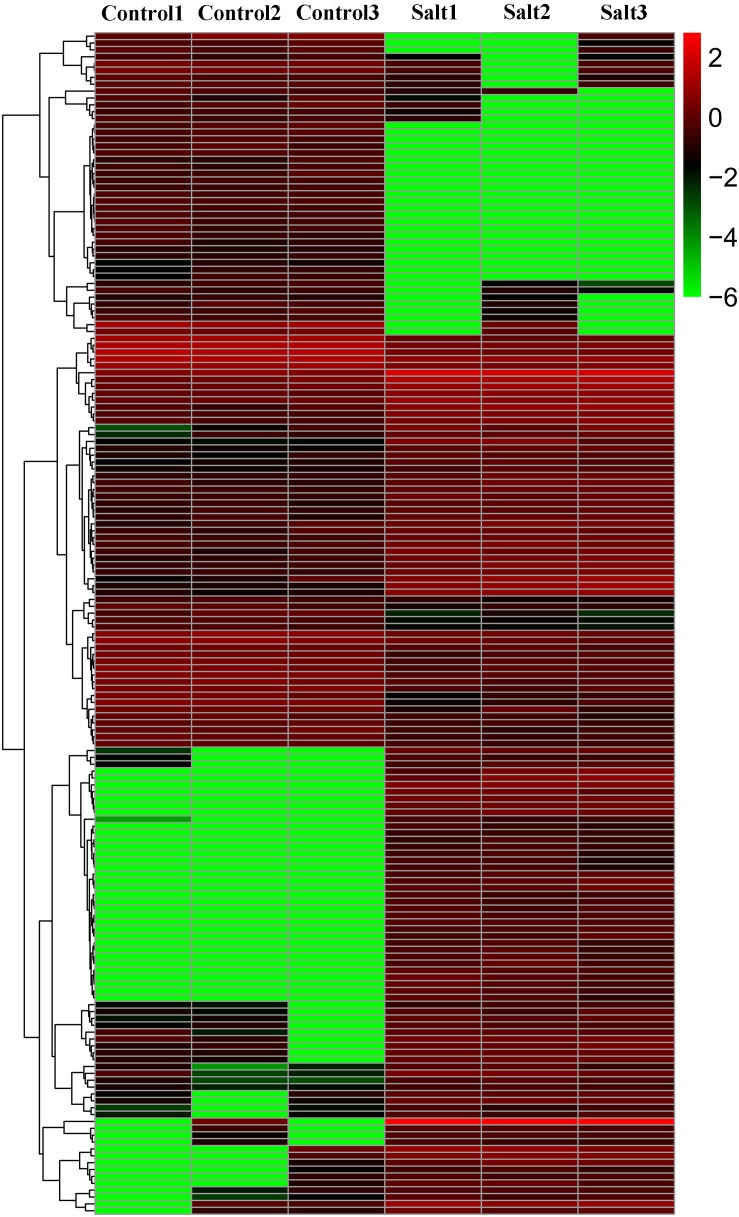
Cluster heat map of differentially expressed lncRNAs (DE-lncRNAs) in control and NaCl-treated tea plants.

In this study, 172 DE-lncRNAs in tea plants were identified by DESeq using the FPKM value, including 101 upregulated and 71 downregulated lncRNAs (Figure 3 and Supplementary Table S1), suggesting that these lncRNAs may function in response to salt stress.

**FIGURE 4:**
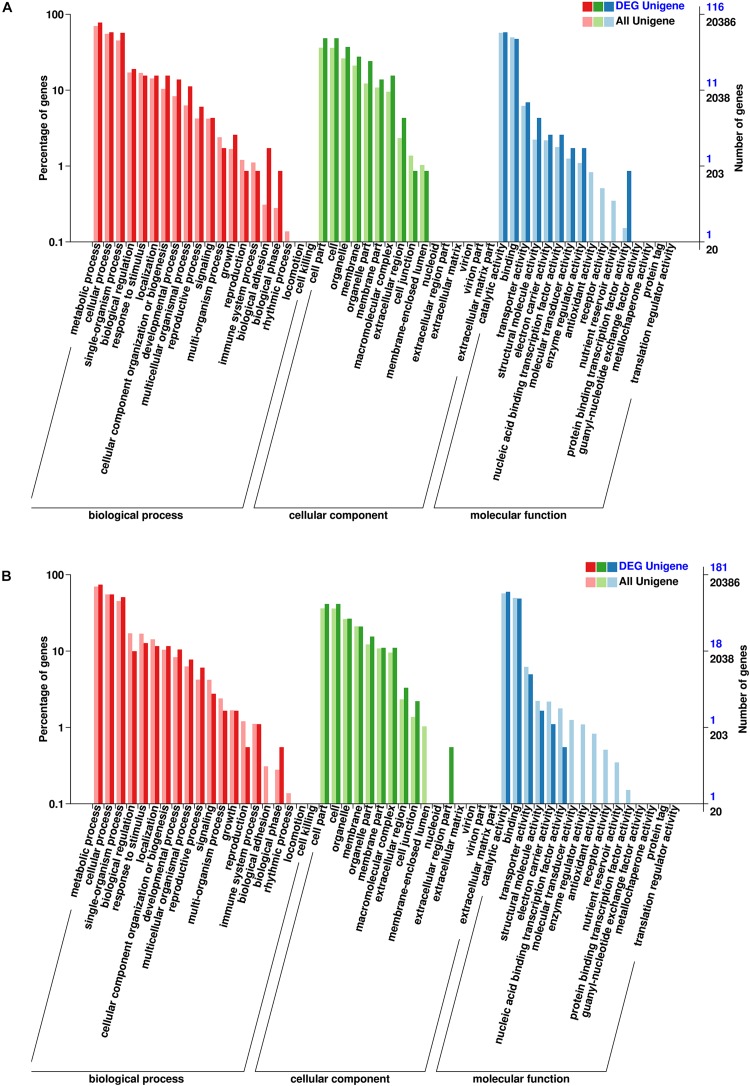
**(A)** Gene Ontology (GO) enrichment of cis-target genes of the DE-lncRNAs. **(B)** GO enrichment of trans-target genes of the DE-lncRNAs.

**FIGURE 5:**
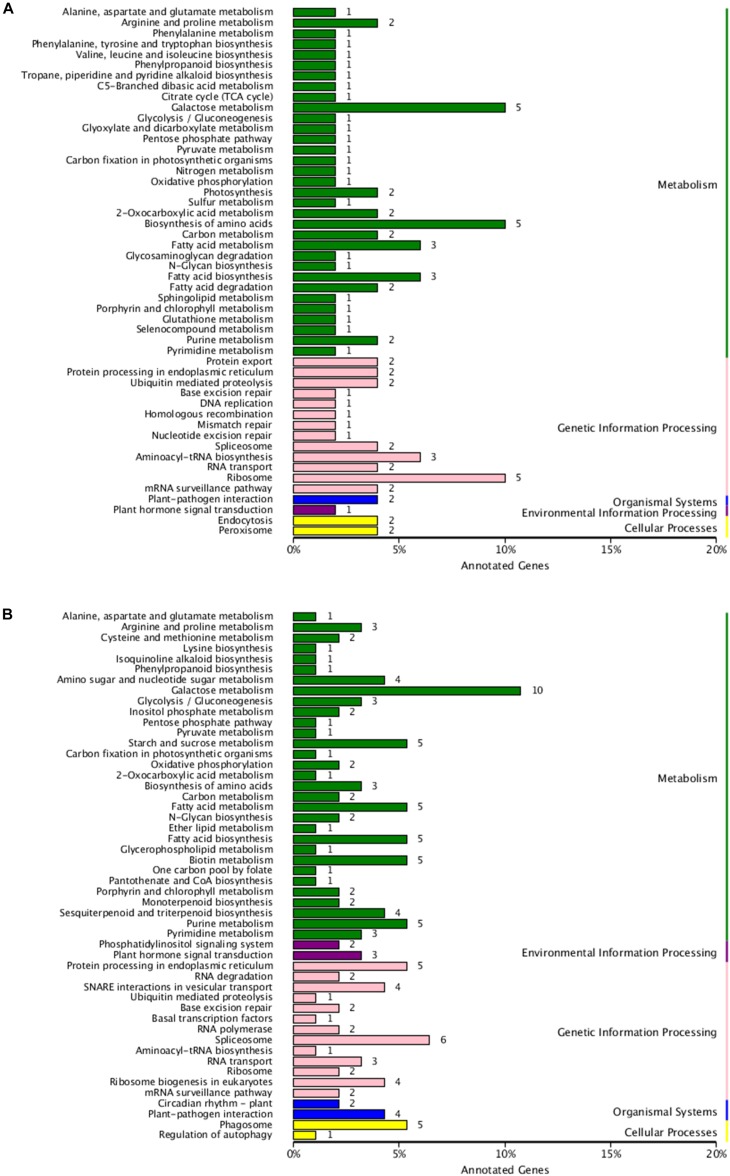
**(A)** Kyoto Encyclopedia of Genes and Genomes (KEGG) enrichment of cis-target genes of the DE-lncRNAs. **(B)** KEGG enrichment of trans-target genes of the DE-lncRNAs.

To analyze the potential functions of these lncRNAs, the predicted cis-target genes encoding genes 100 kb upstream or downstream of these DE-lncRNAs and trans-target genes predicted by complementary pairing of bases were searched and annotated. In total, 250 cis- and 421 trans-target genes of these DE-lncRNAs were predicted (Supplementary Tables S2, S3). Furthermore, GO and KEGG enrichment analyses were used to investigate the potential functions of the cis- and trans-target genes, respectively. As shown in Figures 4A,B, 116 cis-target and 181 trans-target genes were annotated in the GO database. For both cis- and trans-target genes, “Cell part” (GO: 0005623) was the largest subcategory in the cellular component category; “Catalytic activity” (GO: 0003824) in the molecular function category, and “Metabolic process” (GO: 0008152) in the biological process category were the most abundant terms. In addition, 50 cis- and 93 trans-target genes were assigned to 50 and 63 KEGG pathways, respectively (Figures 5A,B), which mainly included “Galactose metabolism” (ko00052), “Biosynthesis of amino acids” (ko01230), and “Ribosome” (ko03010). The results showed that these pathways may play important roles in the salt-stress response of tea plants.

### Interactions of DE-lncRNAs With mRNAs

**FIGURE 6:**
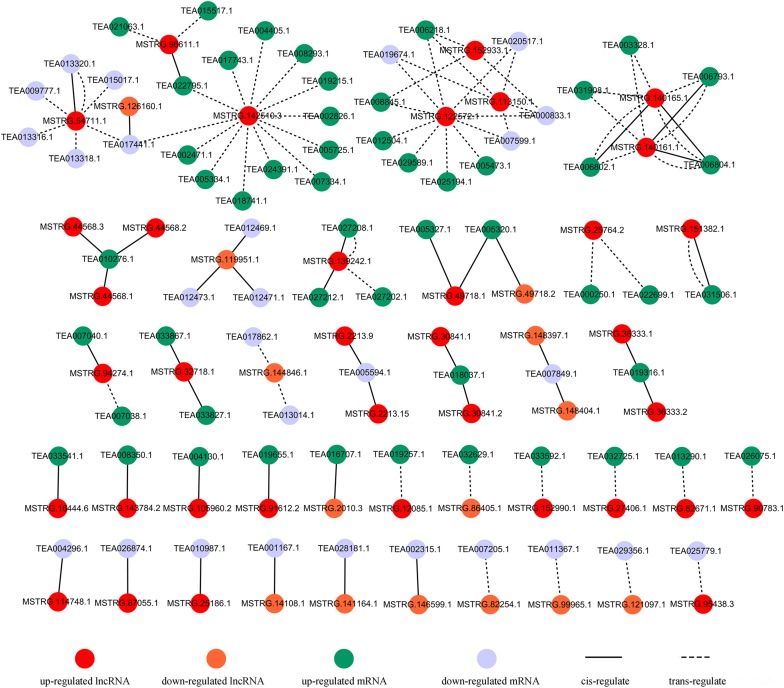
Predicted interaction networks among lncRNAs and mRNAs; details are shown in Supplementary Data Sheet S1: Tables S3, S4.

Recent studies in plants have found that lncRNAs may co-express with nearby coding genes, thereby regulating the downstream targets of the coding genes and exerting biological functions (Cui et al., 2017, 2018). Interestingly, in this study, 42 differentially expressed coding genes spaced less than 100 kb away from 35 DE-lncRNAs were identified (Figure 6 and Supplementary Data Sheet S1: Table S3). These coding genes were annotated as auxin-responsive protein (*TEA005327.1*), Ca^2+^-transporting ATPase 13 (*TEA027212.1*), vacuole membrane protein KMS1-like (*TEA033827.1*), etc. In addition, 67 differentially expressed coding genes were predicted to be trans-target genes of 23 DE-lncRNAs (Figure 6 and Supplementary Data Sheet S1: Table S4). These trans-target genes were annotated as galactinol synthase (GOLS) 2 (*TEA006804.1*), WRKY TF 31 isoform X2 (*TEA005334.1*), ethylene-responsive TF ABR1 (*TEA015017.1*), MYC2 TF (*TEA000833.1*), etc. These differentially expressed mRNAs may interact with corresponding lncRNAs and participate in the salt-stress response through various pathways.

### LncRNAs Participate in Galactinol Synthase, Calcium Signaling Pathway, and Interact With Transcription Factors Under Salt Stress

Synthesis of sugars and signal transduction pathways are crucial participants in regulatory networks of plant responses to salt stress. As presented in Table 2, eight cis/trans target genes GOLS 2 were identified to be upregulated under salt stress. Similarly, six cis/trans target genes related to the Ca^2+^ signaling pathway were identified, including genes encoding calcium-transporting ATPase, calmodulin-interacting protein, Ca^2+^-dependent protein kinases (CDPKs), and CBL-interacting protein kinases (CIPKs). Most of these genes were upregulated except CIPK 23 (TEA032544.1).

**TABLE 2:** LncRNA target genes involved in galactinol synthase and calcium signaling pathway.

Target gene	Regulation	Annotation	Interaction mode
TEA006791.1	Up	Galactinol synthase 2	Cis/trans
TEA006802.1	Up	Galactinol synthase 2	Cis/trans
TEA006793.1	Up	Galactinol synthase 2	Cis/trans
TEA006811.1	Up	Galactinol synthase 2	Cis/trans
TEA006804.1	Up	Galactinol synthase 2	Cis/trans
TEA011903.1	Up	Galactinol synthase 2	Trans
TEA031908.1	Up	Galactinol synthase 2	Trans
TEA003328.1	Up	Galactinol synthase 2	Trans
TEA027212.1	Up	Calcium-transporting ATPase 13	Cis
TEA020781.1	Up	Calcium-dependent protein kinase 16	Cis
TEA027208.1	Up	Calcium-transporting ATPase 13	Cis/trans
TEA023759.1	Up	Calmodulin-interacting protein 111	Trans
TEA019257.1	Up	Calcium-dependent protein kinase 28	Trans
TEA032544.1	Down	CBL-interacting protein kinase 23	Trans

In addition, many TF families play vital roles in regulating plant resistance mechanisms under abiotic stress. In the present study, 23 DE-lncRNA target genes were identified, including HSF, GATA, MADS-box, bHLH, ERF, WRKY, ICE, LHY, and MYC TFs. Except all of the bHLH, LHY and MYC TFs and some of the TFs in GATA and MADS families were downregulated; the rest of the TFs were upregulated (Table 3).

**TABLE 3:** LncRNA target transcription factors.

Target gene	Regulation	Interaction mode	Annotation
TEA022795.1	Up	Cis/trans	HSF
TEA009409.1	Down	Cis	GATA transcription factor 15
TEA013465.1	Down	Cis	GATA transcription factor 28
TEA006583.1	Up	Cis	MADS-box transcription factor
TEA011370.1	Down	Cis	bHLH70
TEA008966.1	Up	Cis	MADS-box transcription factor 23
TEA002032.1	Up	Cis	GATA transcription factor 24
TEA017704.1	Up	Cis	ERF118
TEA002471.1	Up	Trans	WRKY7
TEA013512.1	Up	Trans	ICE1
TEA011367.1	Down	Trans	LHY
TEA007038.1	Up	Trans	ERF4
TEA024930.1	Down	Trans	bHLH155
TEA008962.1	Down	Trans	MADS-box transcription factor 23
TEA015017.1	Up	Trans	ERF110 (ABR1)
TEA022018.1	Down	Trans	bHLH74
TEA007969.1	Up	Trans	WRKY transcription factor 31
TEA010590.1	Down	Trans	bHLH transcription factor 1
TEA010741.1	Down	Trans	MYC1
TEA005334.1	Up	Trans	WRKY transcription factor 31
TEA021401.1	Up	Trans	ERF4
TEA000833.1	Up	Trans	MYC2
TEA005142.1	Up	Trans	WRKY6

### Interactions of DE-lncRNAs With miRNAs

In total, 12 lncRNAs were predicted to be target mimics of 17 known mature miRNAs in *C. sinensis* (Table 4). Interestingly, four miRNAs, *lja-miR7539*, *csn-miR156h*, *ath-miR156i*, and *csn-miR156h*, were predicted to be targeted by more than one lncRNA. Similarly, five lncRNAs, *MSTRG.56302.5*, *MSTRG.116911.1*, *MSTRG.36615.10*, *MSTRG.55773.2*, and *MSTRG.92784.12*, were predicted to be targets of more than one miRNA. Thus, it can be inferred that these lncRNAs and miRNAs may form a complex regulatory network in response to salt stress.

**TABLE 4:** Putative targets of lncRNAs for miRNAs.

miRNA Acc.	Target Acc.	Expect	UPE	miRNA-aligned fragment	Target-aligned fragment	Inhibition
ath-miR156i	MSTRG.56302.5	1	11.063	GAGAGAGAGAGAGAGAGCAG	UUUCUCUCUCUCUCUCUCUC	Cleavage
csn-miR156h	MSTRG.116911.1	1	4.055	UGAGAGAGAGAGAGAGAGCAU	CCCCUCUCUCUCUCUCUCUCA	Cleavage
gma-miR1533c	MSTRG.118391.1	1	10.118	AAAAUAAAAAUAAUAAUAA	UUAUUUUUAUUUUUAUUUU	Cleavage
lja-miR7539	MSTRG.36615.10	1	19.202	GAGAGAGAGAGAGCGAGAGG	CCUCUCUCUCUCUCUCUCUC	Cleavage
	MSTRG.56302.5	1	10.734	GAGAGAGAGAGAGCGAGAGG	UCUCUCUCUCUCUCUCUCUC	Cleavage
	MSTRG.55773.2	1	1.11	GAGAGAGAGAGAGCGAGAGG	UCUCUCUCUCUCUCUCUCUC	Cleavage
	MSTRG.92784.12	1	6.259	GAGAGAGAGAGAGCGAGAG	CUCUCUCUCUCUCUCUCUC	Cleavage
ath-miR426	MSTRG.106114.4	1.5	5.662	AUUUGGAAAAGGAAAGAGAAAAG	UUUUUCUUUUUUCUUUUCUAAAU	Cleavage
ath-miR5021	MSTRG.26810.3	1.5	6.423	AGAGAAGAAGAAGAAGAAAA	UCUUCUUCUUCUUCUUCUUU	Cleavage
ath-miR5998b	MSTRG.143628.1	1.5	7.673	UUAGUUUUUGUUUUGUUUUGU	AAAAAACAAAACAAAAACAAA	Cleavage
csn-miR156h	MSTRG.92784.12	1.5	6.259	UGAGAGAGAGAGAGAGAGCA	CUCUCUCUCUCUCUCUCUCG	Cleavage
	MSTRG.56302.5	1.5	6.06	UGAGAGAGAGAGAGAGAGCAU	UCUCUCUCUCUCUCUCUCUCG	Cleavage
lja-miR7539	MSTRG.91236.3	1.5	7.404	GAGAGAGAGAGAGCGAGAGG	ACUCUCUCUUUCUCUCUCUC	Cleavage
ppe-miR6281	MSTRG.92784.12	1.5	6.259	AUGAGAGAGAGAGAGAGUGAG	CUCUCUCUCUCUCUCUCUCGU	Cleavage
ath-miR156i	MSTRG.36615.10	2	17.653	GAGAGAGAGAGAGAGAGCAG	GCCCUCUCUCUCUCUCUCUC	Cleavage
	MSTRG.55773.2	2	1.11	GAGAGAGAGAGAGAGAGCAG	CAUCUCUCUCUCUCUCUCUC	Cleavage
	MSTRG.116911.1	2	4.044	GAGAGAGAGAGAGAGAGCAG	CCCCUCUCUCUCUCUCUCUC	Cleavage
ath-miR5021	MSTRG.88836.3	2	5.697	AGAGAAGAAGAAGAAGAAAA	CUAUGUUCUUCUUCUUCUCU	Cleavage
csn-miR156h	MSTRG.36615.10	2	18.775	UGAGAGAGAGAGAGAGAGCAU	GCCCUCUCUCUCUCUCUCUCU	Cleavage
	MSTRG.55773.2	2	1.11	UGAGAGAGAGAGAGAGAGCAU	CAUCUCUCUCUCUCUCUCUCU	Cleavage
lja-miR7539	MSTRG.116911.1	2	4.044	GAGAGAGAGAGAGCGAGAGG	CCCCUCUCUCUCUCUCUCUC	Cleavage
mtr-miR2586a	MSTRG.123314.2	2	18.404	AGAGGUGUCCGUGCUUCAU	AUGAAGCAUGGACACAUCU	Cleavage
ppe-miR6281	MSTRG.116911.1	2	4.06	AUGAGAGAGAGAGAGAGUGAG	CCCUCUCUCUCUCUCUCUCAC	Cleavage
ptc-miR6462a	MSTRG.56302.5	2	5.5	UCUCUUUUGCAUUUUUGCUGCC	GAGAGCAAAGAUGAAGAAGAGA	Translation

### LncRNA *MSTRG.139242.1* May Interact With Its Nearby mRNA*TEA027212.1*

**FIGURE 7:**
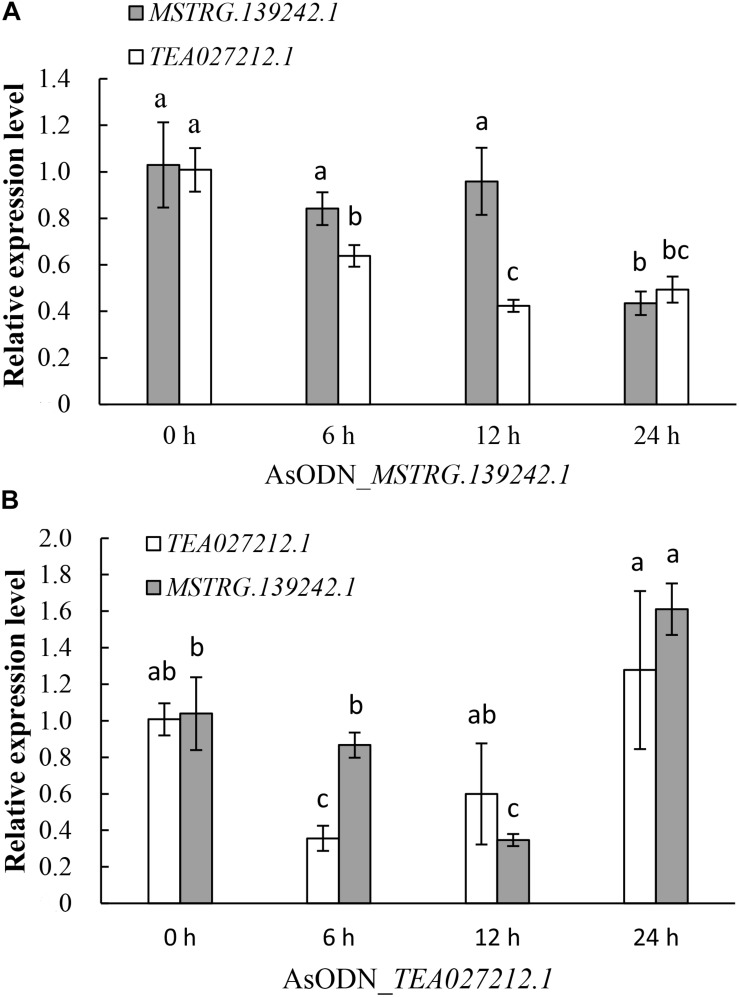
Quantitative real-time PCR (qRT-PCR) analysis of the lncRNA *MSTRG.139242.1* and its target mRNA*TEA027212.1*, using tea leaves treated with AsODN_*MSTRG.139242.1* and AsODN_*TEA027212.1* for 0, 6, 12, and 24 h, respectively. Different letters in the columns represent a significant difference in the qRT-PCR results between the control and antisense oligodeoxynucleotide (AsODN)-treated individuals (*P* < 0.05).

Among all of the target genes of the DE-lncRNAs, we found an upregulated coding gene, *TEA027212.1* (Scaffold765 1270655–1272793), which was annotated as Ca^2+^-transporting ATPase 13, that was located only 5,911 bp adjacent to the upregulated lncRNA *MSTRG.139242.1* (Scaffold765 1278704–1412939) according to the tea tree genome. To verify their expression relationship, a gene suppression assay in tea plants using AsODNs was conducted with *MSTRG.139242.1* and *TEA027212.1*. qRT-PCR results showed that after gene suppression of lncRNA *MSTRG.139242.1*, its expression level was not significantly reduced until 12–24 h, but *TEA027212.1*expression level was significantly reduced from 0 to 12 h (Figure 7A). However, gene suppression of *TEA027212.1* from 0 to 6 h significantly reduced *MSTRG.139242.1* transcript levels from 6 to 12 h (Figure 7B). These results indicate that the lncRNA *MSTRG.139242.1* may be regulated by its nearby coding gene, *TEA027212.1*, and may be involved in Ca^2+^ transport in response to salt stress in tea plants.

### qRT-PCR Validation

**FIGURE 8:**
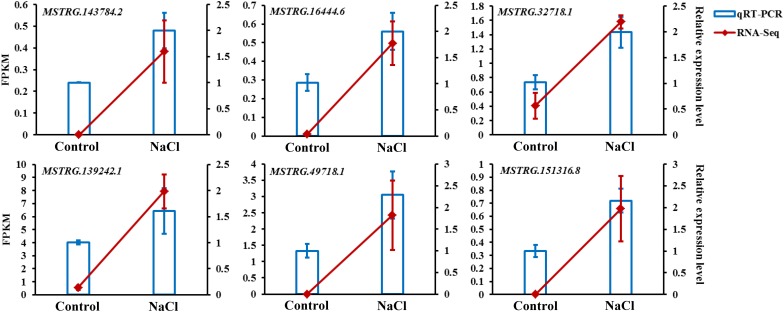
qRT-PCR validation of six DE-lncRNAs selected from the RNA-Seq transcriptome. The expression levels of the RNA-Seq results were calculated using the FPKM value presented on the left axis; qRT-PCR relative expression levels were calculated using the 2^– ΔΔ*Ct*^ method presented on the right axis; the values shown are the means ± standard deviation of *n* = 3 replicates.

To confirm the reliability of the expression levels of lncRNAs obtained from the RNA-Seq transcriptome, six DE-lncRNAs, *MSTRG.143784.2*, *MSTRG.16444.6*, *MSTRG.32718.1*, *MSTRG.139242.1*, *MSTRG.49718.1*, and *MSTRG.151316.8*, were randomly selected for qRT-PCR validation. As shown in Figure 8, the expression levels of these lncRNAs closely corresponded to the transcript level estimated from the sequence data, which indicates the reproducibility and accuracy of the RNA-Seq results.

### Data Deposition

The sequencing data were deposited in the NCBI Sequence Read Archive (SRA) database^13^ under accession number SRP107589. LncRNA sequences identified are attached in Supplementary Table S4.

## Discussion

Salt stress is an important factor that affects the growth of plants, including tea plants, under natural conditions, and the mechanism of the response to salt stress and salt stress-resistance breeding have been studied for a long period. Over the past decade, a number of genes involved in salt stress have been identified and verified, in addition to their regulatory pathways that mediate the transduction of stress signals and the process of the salt-stress response, such as the Ca^2+^ signaling pathway, ABA pathway, and MAPK cascade (Boudsocq and Sheen, 2009; Cheong and Kim, 2010; Yoshida et al., 2014; Abbasi et al., 2016; Zhu, 2016). In tea plants, many coding genes, such as *AQP*, *VQ*, and *SnRK2*, participate in the salt-stress response (Yue et al., 2014; Guo et al., 2018; Zhang et al., 2018). However, a few studies have been performed on the identification and mechanism of salt stress-related non-coding RNAs, especially lncRNAs, although lncRNAs have been proven to be involved in many biological processes in plants (Ponjavic et al., 2007; Ponting et al., 2009). In this study, we systematically identified tea plant lncRNAs based on the latest tea tree genome to find lncRNAs associated with salt stress and preliminarily analyzed their possible interactions with mRNAs or miRNAs. A number of lncRNAs were identified for the first time to be specifically expressed under high concentrations of NaCl and involved in the stress response.

A total of 16,452 candidate lncRNAs from six tea leaf samples were identified in this study. Similar to the lncRNAs identified in other species (Liu et al., 2012; Shuai et al., 2014; Zhu et al., 2015; Tian et al., 2016; Ma et al., 2018), the average length of lncRNAs in tea plants was much shorter than that of mRNAs, and most lncRNAs (66.9%) were lincRNAs. In some plants, functional lncRNAs that were differentially expressed under specific conditions were identified using transcriptome sequencing, and the functions were validated. For example, Zhu et al. (2015) identified 490 lncRNAs that were significantly upregulated in tomato ripening mutants using RNA-Seq, and two novel lncRNAs, *lncRNA1459* and *lncRNA1840*, were proven to function in the delay of fruit ripening by gene silencing and mutagenesis (Li et al., 2018). Ding et al. (2014) conducted RNA-seq of salt-treated *Arabidopsis* seedlings, and Qin et al. (2017) obtained a putative lncRNA named *DRIR* that was later proven to increase tolerance to drought and salt stress. Similarly, 172 lncRNAs with various functions were found to be differentially expressed under NaCl stress in our results. Thus, these lncRNAs are involved in the salt-stress response of tea plants, and the results can provide candidate genes for salt tolerance studies in tea plants.

Previous studies have reported that lncRNAs play important roles in a variety of biological processes in response to stress by acting directly or indirectly on functional genes in plants (Wu et al., 2013; Fatica and Bozzoni, 2014; Wang et al., 2017). Currently, the interactions between lncRNAs, mRNAs, and miRNAs are the main research hotspots. Thus, in this study, the interactions between lncRNAs and mRNAs, and between lncRNAs and miRNAs were predicted.

To the best of our knowledge, lncRNAs can negatively or positively regulate the expression of protein-coding genes by acting in cis or in trans; lncRNAs work in cis when their target genes are on the same chromosome and within a close distance, and lncRNAs work in trans when they affect genes on other chromosomes (Kornienko et al., 2013). In addition, recent studies found that some cis-acting lncRNAs also have the ability to act in trans (Martianov et al., 2007; Schmitz et al., 2010). In this study, possible cis- and trans-target genes were predicted and annotated to explore the possible functions of the DE-lncRNAs. In total, 250 cis- and 421 trans-target genes of these DE-lncRNAs were predicted. GO and KEGG analyses indicated that these lncRNAs participated in various pathways, such as catalytic activity, galactose metabolism, and biosynthesis of amino acids in response to salt stress. Notably, 42 cis- and 67 trans-target genes were differentially expressed under salt stress. Among these target genes, Ca^2+^-transporting ATPase (*TEA027212.1*) was reported to be an important gene in Ca^2+^ signaling under abiotic stress (Wilkins et al., 2016), which suggests that its corresponding lncRNA, *MSTRG.139242.1*, may interact with Ca^2+^-transporting ATPase and may be involved in this process. Thus, a gene suppression assay of *MSTRG.139242.1* and *TEA027212.1* (Ca^2+^-transporting ATPase 13) using AsODNs was conducted and suggested that lncRNA *MSTRG.139242.1* may be regulated by its nearby coding gene, *TEA027212.1*. In a previous study, the tomato lncRNA *lncRNA33732* activated by *WRKY1* induces *RBOH* expression and conferred resistance to *Phytophthora infestans* infection (Cui et al., 2018). In this study, we can speculate that lncRNA *MSTRG.139242.1* may interact with its nearby mRNA TEA027212.1 in response to salt stress. In addition, an auxin-responsive protein (*TEA005327.1*) and ethylene-responsive TF ABR1 (*TEA015017.1*), the target genes of the lncRNAs *MSTRG.49718.1* and *MSTRG.54711.1*, respectively, have been reported to be involved in the auxin, ethylene, and ABA signal transduction pathways in response to abiotic stresses in other plants (Pandey et al., 2005; Jain and Khurana, 2009; Feng et al., 2015), indicating that these two lncRNAs may participate in hormone signal transduction in response to salt stress in tea plants. We speculate that the expression of these target mRNAs may be regulated by their upstream lncRNAs in response to salt stress.

In addition, it is worth noting that many lncRNA target genes were classified as GOLS, calcium signaling, and some crucial TFs. GOLS was reported to be a key enzyme in the synthesis of raffinose family oligosaccharides (RFOs) and catalyzes the condensation of UDP-galactose with myo-inositol to produce galactinol as the sole donor for the synthesis of RFOs. In addition, RFOs are used for the transport and storage of carbohydrates and as compatible solutes for protection against abiotic and biotic stresses (Peters and Keller, 2009; Philippe et al., 2010). Sun Z.B. et al. (2013) have reported that *TsGOLS2* enhances tolerance to high salinity and osmotic stresses in *Arabidopsis thaliana*. Thus, in the present study, lncRNAs targeting the eight upregulated *GOLS* genes may participate in RFO synthesis in response to salt stress. The Ca^2+^ signaling pathway was also reported to mediate plant response to salt stress, which starts with Ca^2+^ transporters such as Ca^2+^-ATPases, Ca^2+^ sensors, and relay proteins such as CaM, CMLs, CDPKs, CBLs, and CIPKs (Wilkins et al., 2016). LncRNAs targeting calcium-transporting ATPase, calmodulin-interacting protein, CDPKs and CIPKs in this study were predicted to be involved in salt-stress response through the Ca^2+^ signaling pathway. TFs have long been an important topic in plant resistance research, and some TF families such as WRKY, bHLH, ERF, GATA, and MYC play significant roles in salt stress adaptation (Lindemose et al., 2013). Here, the results provide many candidate lncRNAs, which may interact with vital TFs and response to salt stress.

On the other hand, lncRNAs can also interact with miRNAs. Some miRNAs have been found to respond to different environmental stresses (Zhang, 2015). Some lncRNAs can bind to miRNAs, thus inhibiting the effect of miRNAs on downstream genes. This type of lncRNA is termed an endogenous target mimic (eTM) (Wu et al., 2013). Jiang et al. (2019) reported that the tomato *lncRNA23468* functions as an eTM to modulate NBS-LRR genes by mimicking miR482b in the tomato–*P. infestans* interaction. In this study, 12 lncRNAs were predicted to be target mimics of 17 known mature miRNAs. Interestingly, five lncRNAs, *MSTRG.56302.5*, *MSTRG.116911.1*, *MSTRG.92784.12*, *MSTRG.36615.10*, and *MSTRG.55773.2*, were predicted to be eTMs of *miR156*. According to previous reports, *miR156* is induced by drought, and salinity stresses in several different plant species specifically regulates downstream *SPL* TFs and affects plant morphology, phase change, and seed germination (Stief et al., 2014). Thus, the five lncRNAs may influence the effect of *miRNA156* on *SPL* in response to salt stress. These results provide candidate lncRNAs for exploring the interactions between lncRNAs and miRNAs in response to salt stress.

## Conclusion

In the present study, genome-wide identification of lncRNAs was performed using high-throughput RNA-Seq and bioinformatics analysis. lncRNAs (16,452) were identified in tea plants, among which, 172 lncRNAs were differentially expressed and responsive to salt stress by cis- or trans-interaction with important coding genes. These lncRNAs may participate in GOLS, calcium signaling pathway, and interact with TFs in response to salt stress. Notably, 35 DE-lncRNAs were predicted to interact with 42 differentially expressed coding genes, which may participate in pathways such as the auxin response, ABA, and Ca^2+^ signal transduction pathways under salt stress. AsODN suppression of the lncRNA *MSTRG.139242.1* and its predicted interacting gene, *TEA027212.1* (Ca^2+^-ATPase 13), indicated that *MSTRG.139242.1* interacts with Ca^2+^-ATPase 13 in the Ca^2+^-transport pathway in response to high salinity in tea plants. In addition, 12 lncRNAs were predicted to be target mimics of 17 known mature miRNAs in *C. sinensis*, thereby affecting the expression of downstream functional genes. This study can provide a source of lncRNAs and benefit an in-depth understanding of the function and regulatory mechanisms in tea plant response to salt stress.

## Data Availability Statement

All datasets generated for this study are included in the article/Supplementary Material.

## Author Contributions

YYu and YYa designed the experiments. SW and YZ performed the experiments and data analysis. SW and YZ wrote the manuscript. WW and QX provided valuable advice on the manuscript. LH and MD revised the manuscript. All authors discussed the results and contributed to the manuscript.

## Conflict of Interest

The authors declare that the research was conducted in the absence of any commercial or financial relationships that could be construed as a potential conflict of interest.

## Footnotes

1 http://ccb.jhu.edu/software/hisat2/index.shtml

2 https://ccb.jhu.edu/software/stringtie/index.shtml

3 http://ccb.jhu.edu/software/stringtie/gffcompare.shtml

4 http://cpc.cbi.pku.edu.cn/

5 http://www.ncbi.nlm.nih.gov/pubmed/23892401

6 http://lilab.research.bcm.edu/cpat/

7 http://pfam.xfam.org/

8 http://cole-trapnell-lab.github.io/cufflinks/cuffcompare/

9 http://www.bioconductor.org/packages/release/bioc/html/DESeq.html

10 https://plantgrn.noble.org/psRNATarget/

11 http://cytoscapeweb.cytoscape.org/

12 http://sfold.wadsworth.org/cgi-bin/soligo.pl

13 http://www.ncbi.nlm.nih.gov/Traces/sra

## Supplementary Material

TABLE S1 Statistics of DE-lncRNAs.

TABLE S2 Statistics of cis-target genes of DE-lncRNAs.

TABLE S3 Statistics of trans-target genes of DE-lncRNAs.

TABLE S4 LncRNA sequences identificated in this study.

DATASHEET S1 Sequences for AsODN assay, primers used in qRT-PCR and differentially expressed target genes.
