# Supplementary material for: Integrated Analysis of Long Non-coding RNAs (lncRNAs) and mRNAs Reveals the Regulatory Role of lncRNAs Associated With Salt Resistance in Camellia sinensis
Source: Front Plant Sci. 2020 Mar 19;11:218. doi: 10.3389/fpls.2020.00218 (PMC7096555; doi:10.3389/fpls.2020.00218)
Supplement: DATASHEET S1 — Sequences for AsODN assay, primers used in qRT-PCR and differentially expressed target genes. [file Data_Sheet_1.docx]

**Table S1 Sequences of lncRNA and mRNA for AsODN assay and selected ODNs**

|  | Sequence |
| --- | --- |
| *MSTRG.139242.1* | CAGTGTCAAGAGCCAAAGCTATGCCTTCAAGTCCTCCAAGTTGAGCAAGATCTTCTACATTCTTTTGCTTCATGAGTTGCGTGAGGCTGTTTTGATTAATCTTGTAGGAGTTGGGTGGGTCTAGGTTGACATTGAGGGCAGTGTAGGATGGTGAACGTTGGTATTTGGAGTGTTTGTTTGCTACGATTTGTTTGAAATGAGAGTACAAGGCTCTTGAACAGTAGATAGTTGAGAAAGCCAAGCGCCATCTCTTGATGGGTATGCTAAGGGTGGTGATTGGCACGTTGTGTAAAGGGTCGATACACTGCATGTTTGCTTGTATTATGGTAGACATGGTTAGTAAAAAAGAATGAAAATAGGTGGAACAGAGATGGGAAATGATGGGTTTTCAATAGCACAATGTAATGCAGAATCAACAATCGAGAATGA |
| *TEA027212.1* | ATGAGCACAATCAGCCGCGACACCAACGAGCAAACTCCTTTACAAGCCCGGCTCAACAAACTAACCTCGGCCATAGGCAAGGTTGGTTTGGTTGTTGCTTTCCTAGTTCTAGTGGTGTTGTTGGTTCGGTATTTCACAGGCAATACAAAAGATGCAAATGGAAATACAGAGTTCAATGGAAGCAAGACAAAAGTTAATGACATTATTAATTCTGTTGTGGGGATTGTAGCTGCTGCAGTAACTATTGTTGTTGTTGCGATTCCTGAAGGCTTGCCATTGGCTGTCACACTTACTCTTGCATATTCGATGAAGAGAATGATGGCTGATCAAGCCATGGTTCGAAAGCTTTCAGCTTGCGAAACAATGGGCTCTGCCACTACTATATGTACTGACAAAACAGGCACACTTACACTGAATGAAATGACAGTCACAAAGTTTTGGCTAGGCCAAGATTGCATAGAAGGAAGAGGTTCAAGTACCTCAATTGCAGCCAATGTTCTTGAATTGCTCTGCCAAGGAATAGCTTTGAACACAACTGGCAGTGTCTACAAGTGCACTTCAGGATCCGAATTTGAGTTATCGGGTAGTCCCACTGAAAAAGCAATTCTTTCTTGGGGATTCCTAGAATTGAACATGGACATGGCAGTGTTGAAAGAAAATTGTATGGTTCTCCATGTTGAGGCCTTCAATTCGGAGAAGAAACGAAGCGGGGTTTTAATGAAGAGAAAATTTGACAACACGGTCCATGTGCACTGGAAAGGAGCTGCAGAAATGGTACTCGCGATGTGCTCTCATTACTATGATTGTTCTGGAAACATGAAAGTTCTGGGTGATAGTGAAAGAATGAGATTTGATCAAATAATTCAAGGCATGGCTGCTAATAGTCTCCGGTGCATCTCTTTTGCTCATAAGAAAATCTCAGAAGCAGAGCATGCAGCAGGAGAAGATGATCAGAAGCTAAGTGAAACCTGCTTAGCCCTATTGGGGATGGTGGGTCTGAAAGACCCATGTCGACCCGGTGTGAAGAAAGCTGTGGAAGATTGTCAATTAGCTGGAGTAAGCGTCAAAATGATCACTGGCGATAATATTTTCACTGCAAGAGCAATTGCCACCGAATGTGGGATACTCAGGCCAAATCAGGACATAGGCAATGAAGCAGTAATAGAAGGTGTGAAATTTCGAAACTACACACCTGAAGAGCGAATTGAAAGAGTTGAAAAAATTTGTGTGATGGCCAGATCTTCCCCCTTGGACAAGCTTCTAATGGTGCAATGCTTGAAACAGAAAGGTCATGTTGTCGCGGTCACTGGGGATGGCACGAATGATGCACCGGCCTTGAAGGAAGCAGACATAGGCCTTTCTATGGGGATTCAGGGGACTGAAGTGGCGAAAGAGAGCTCGGACATTGTCATATTGGATGATAACTTTGCTTCTGTAACCACGGTCTTGTGTTGGGGTAGATGTGTTTATAACAACATCCAAAAATTCATCCAGTTTCAACTCACTGTGAATGTAGCTGCACTTGTCATCAATTTTGTGGCAGTTGTTTCAGCCGGTGAAGTTCCTCTAACCACGGTCCAGCTTCTGTGGGTGAATTTGATCATGGACACATTAGGTGCTCTGGCTCTAGCCACAGAGAAGCCCACAAAGGAGCTCATGACAAAGCTGCCGGTGGGTCGAACCGAGCCACTTATCACCAACATCATGTGGAGGAACCTGCTAGCTCAAGCTCTGTACCAAATAGCAGTCCTACTGGCCTTGCAGTTCAGAGGCGAATCAATATTGGGGGTGAATCAGAGTGTGAACAATACTTTGATCTTCAATACTTTTGTGCTTTGCCAAGTCTTCAATGAATTCAATGCACGAAAGCTTGAGAAGAAGGATGTGTTTGAAGGGATATTGAGGAACAGGTTGTTTGTGGGGATCATTGGGATAACCATTGTTCTTCAAGTGGTGATGGTGGAGTTTCTGAAGAAGTTTGCAGGTACAGAGAGGTTGAGCTGGGGGCAATGGGGAGTGTGTGTTGGAATTGCTTCTGTGTCTTGGCCCATTGGTTGGGTTTTCAAGTGCATTCCTGTACCAGAGAAGCCATTTTGCAGCTATCTCAAATGGCAGAACTTGAAATGCGGTTGCAAATAA |
| *MSTRG.139242.1* ODN-1 | CAGTGTATCGACCCTTTACA |
| *MSTRG.139242.1* ODN-2 | CCATCATTTCCCATCTCTGT |
| *TEA027212.1* ODN-1 | CCGCTTCGTTTCTTCTCCGA |
| *TEA027212.1* ODN-2 | TGGACCGTGGTTAGAGGAAC |
| *TEA027212.1* ODN-3 | ATCCTTCTTCTCAAGCTTTC |
| nonsense control | GGCGGCTAACGCTTCGACTA |

**Table S2 Primer sequences used in qRT-PCR**

| Gene ID | Primer F (5'-3') | Primer R (5'-3') |
| --- | --- | --- |
| *MSTRG.143784.2* | ACTCGTGAACCCTCTCAA | GACTCCACACATGAACTGTAA |
| *MSTRG.16444.6* | CAACCACAGCAACAACAAAG | GGAGGATGAGAAAGGCAAAG |
| *MSTRG.32718.1* | GCTCTTGTGTAGAGGCAATAA | ATTGAGGCTCCTTCTTGTATG |
| *MSTRG.139242.1* | GATTGGCACGTTGTGTAAAG | AACCCATCATTTCCCATCTC |
| *MSTRG.49718.1* | AGGGTCTGAGATTAGCATAGT | ACCACCACCACCTCTATAA |
| *MSTRG.151316.8* | ATAGCGAATACAAGTGAGATGG | CACAGACTGCACAGGAATAC |
| *TEA027212.1* | CCTTGAAGGAAGCAGACATAG | CAAGACCGTGGTTACAGAAG |
| *Csβ-Actin* | GCCATCTTTGATTGGAATGG | GGTGCCACAACCTTGATCTT |

**Table S3** Differentially expressed cis-target genes of DE-lncRNAs

| LncRNA_ID | regulated | Cis-target gene（100kb） | regulated | annotation |
| --- | --- | --- | --- | --- |
| MSTRG.151382.1 | up | TEA031506.1 | up | hypothetical protein CCACVL1_15597 |
| MSTRG.2010.3 | up | TEA016707.1 | down | protein ALTERED XYLOGLUCAN 4 |
| MSTRG.44568.1 | up | TEA010276.1 | up | Kinesin domain-containing protein/DAP_epimerase domain-containing protein |
| MSTRG.44568.3 | up | TEA010276.1 | up | Kinesin domain-containing protein/DAP_epimerase domain-containing protein |
| MSTRG.94274.1 | up | TEA007040.1 | up | protein OBERON 4 |
| MSTRG.114748.1 | down | TEA004296.1 | up | ubiquitin-conjugating enzyme E2-23 kDa isoform X1 |
| MSTRG.119951.1 | down | TEA012473.1 | down |  |
|  |  | TEA012471.1 | down | Agglutinin-like protein ALA1, putative isoform 2 |
|  |  | TEA012469.1 | down |  |
| MSTRG.126160.1 | down | TEA017441.1 | down | PROLINE-RICH protein 4 |
| MSTRG.14108.1 | down | TEA001167.1 | down | putative tea geometrid larvae-inducible protein |
| MSTRG.141164.1 | down | TEA028181.1 | down | unnamed protein product, partial |
| MSTRG.146599.1 | down | TEA002315.1 | down | tropinone reductase homolog |
| MSTRG.148397.1 | down | TEA007849.1 | down |  |
| MSTRG.2213.9 | down | TEA005594.1 | up | uncharacterized protein At1g01500-like |
| MSTRG.25186.1 | down | TEA010987.1 | up | uncharacterized protein LOC104228664 |
| MSTRG.87055.1 | down | TEA026874.1 | up | EG45-like domain containing protein |
| MSTRG.139242.1 | Up | TEA027212.1 | up | calcium-transporting ATPase 13, plasma membrane-type |
|  |  | TEA027208.1 | up | putative calcium-transporting ATPase 13, plasma membrane-type |
| MSTRG.140161.1 | Up | TEA006793.1 | up | galactinol synthase 2-like |
|  |  | TEA006804.1 | up | galactinol synthase 2 |
| MSTRG.16444.6 | up | TEA033541.1 | up | INO80 complex subunit B like |
| MSTRG.30841.1 | up | TEA018037.1 | up | Oxysterol-binding protein |
| MSTRG.30841.2 | up | TEA018037.1 | up | Oxysterol-binding protein |
| MSTRG.32718.1 | Up | TEA033867.1 | up | vacuole membrane protein 1-like |
|  |  | TEA033827.1 | up | vacuole membrane protein KMS1-like |
| MSTRG.49718.1 | Up | TEA005320.1 | down | elongation factor G-2, chloroplastic |
|  |  | TEA005327.1 | up | Auxin-responsive protein |
| MSTRG.96611.1 | up | TEA022795.1 | up | Heat shock factor (HSF)-type, DNA-binding |
| MSTRG.148404.1 | down | TEA007849.1 | down |  |
| MSTRG.2213.15 | down | TEA005594.1 | up | uncharacterized protein At1g01500-like |
| MSTRG.54711.1 | down | TEA013320.1 | down | putative allergen I1 |
| MSTRG.105960.2 | up | TEA004130.1 | up | glutathione S-transferase |
| MSTRG.140165.1 | Up | TEA006802.1 | up | galactinol synthase 2 |
|  |  | TEA006804.1 | up | galactinol synthase 2 |
| MSTRG.143784.2 | up | TEA008350.1 | up | dynein light chain LC6, flagellar outer arm |
| MSTRG.36333.1 | up | TEA019316.1 | up | Leucine-rich repeat family protein |
| MSTRG.44568.2 | up | TEA010276.1 | up | Kinesin domain-containing protein/DAP_epimerase domain-containing protein |
| MSTRG.49718.2 | up | TEA005320.1 | down | elongation factor G-2, chloroplastic |
| MSTRG.36333.2 | up | TEA019316.1 | up | Leucine-rich repeat family protein |
| MSTRG.91612.2 | up | TEA019655.1 | up | uncharacterized protein LOC100258555 |
| MSTRG.2213.7 | down | TEA005594.1 | up | uncharacterized protein At1g01500-like |

**Table S4** Differentially expressed trans-target genes of DE-lncRNAs

| LncRNA_ID | regulated | trans-target gene（100kb） | regulated | annotation |
| --- | --- | --- | --- | --- |
| MSTRG.139242.1 | up | TEA027202.1 | up |  |
|  |  | TEA027208.1 | up | putative calcium-transporting ATPase 13, plasma membrane-type |
| MSTRG.140165.1 | up | TEA006793.1 | up | galactinol synthase 2-like |
|  |  | TEA006804.1 | up | galactinol synthase 2 |
|  |  | TEA031908.1 | up | galactinol synthase 2 |
|  |  | TEA006802.1 | up | galactinol synthase 2 |
|  |  | TEA003328.1 | up | galactinol synthase 2-like |
| MSTRG.142510.3 | up | TEA008293.1 | up | lipid transfer protein EARLI 1-like |
|  |  | TEA017743.1 | up | pEARLI1-like lipid transfer protein 3 |
|  |  | TEA017441.1 | down | PROLINE-RICH protein 4 |
|  |  | TEA024391.1 | down | UDP-glycosyltransferase 74Y1 |
|  |  | TEA018741.1 | down | PROLINE-RICH protein 4 |
|  |  | TEA002471.1 | up | tRNA-dihydrouridine(16/17) synthase [NAD(P)(+)]-like |
|  |  | TEA002826.1 | down | pollen-specific leucine-rich repeat extensin-like protein 1 |
|  |  | TEA005334.1 | up | probable WRKY transcription factor 31 isoform X2 |
|  |  | TEA004405.1 | up | uncharacterized protein At5g39570-like isoform X5 |
|  |  | TEA005725.1 | up | Uncharacterized protein TCM_036932 |
|  |  | TEA019215.1 | up | hypothetical protein CICLE_v10010435mg, partial |
|  |  | TEA022795.1 | up | Heat shock factor (HSF)-type, DNA-binding |
| MSTRG.151382.1 | up | TEA031506.1 | up | hypothetical protein CCACVL1_15597 |
| MSTRG.152990.1 | up | TEA033592.1 | up |  |
| MSTRG.27406.1 | up | TEA032725.1 | up | dehydrin |
| MSTRG.62671.1 | up | TEA013290.1 | up | auxin-repressed protein |
| MSTRG.90783.1 | up | TEA026075.1 | up | beta-ketoacyl-ACP synthase I |
| MSTRG.144846.1 | down | TEA013014.1 | down | unnamed protein product |
|  |  | TEA017862.1 | down |  |
| MSTRG.54711.1 | down | TEA013318.1 | down | putative allergen I1 |
|  |  | TEA013316.1 | down |  |
|  |  | TEA013320.1 | down | putative allergen I1 |
|  |  | TEA009777.1 | up | arginine decarboxylase |
|  |  | TEA017441.1 | down | PROLINE-RICH protein 4 |
|  |  | TEA015017.1 | up | ethylene-responsive transcription factor ABR1 |
| MSTRG.82254.1 | down | TEA007205.1 | down | salutaridine reductase-like |
| MSTRG.99965.1 | down | TEA011367.1 | down | LHY |
| MSTRG.12085.1 | up | TEA019257.1 | up | calcium-dependent protein kinase 28 |
| MSTRG.94274.1 | up | TEA007038.1 | up | ethylene response factor |
| MSTRG.95438.3 | down | TEA025779.1 | up | hypothetical protein CFOL_v3_31178 |
| MSTRG.140161.1 | up | TEA006793.1 | up | galactinol synthase 2-like |
|  |  | TEA006804.1 | up | galactinol synthase 2 |
|  |  | TEA031908.1 | up | galactinol synthase 2 |
|  |  | TEA006802.1 | up | galactinol synthase 2 |
|  |  | TEA003328.1 | up | galactinol synthase 2-like |
| MSTRG.86405.1 | up | TEA032629.1 | down | uncharacterized protein LOC100256501 isoform X1 |
| MSTRG.121097.1 | down | TEA029356.1 | down | terpene synthase |
| MSTRG.25764.2 | up | TEA000250.1 | up | CLAVATA3/ESR (CLE)-related protein 43 |
|  |  | TEA022699.1 | up | ninja-family protein AFP3 |
| MSTRG.96611.1 | up | TEA015517.1 | up |  |
|  |  | TEA021063.1 | up |  |
| MSTRG.113150.1 | down | TEA006218.1 | up | thioredoxin-like 3-1, chloroplastic |
|  |  | TEA000833.1 | up | MYC2 trancription facor |
|  |  | TEA020517.1 | up | alpha carbonic anhydrase 1, chloroplastic |
|  |  | TEA019674.1 | up | aquaporin PIP2-1-like |
|  |  | TEA007599.1 | up | BOI-related E3 ubiquitin-protein ligase 1-like isoform X3 |
| MSTRG.122572.1 | up | TEA006218.1 | up | thioredoxin-like 3-1, chloroplastic |
|  |  | TEA029589.1 | up | PREDICTED: protein PMR5 |
|  |  | TEA008845.1 | up | probable plastidic glucose transporter 2 |
|  |  | TEA007599.1 | up | BOI-related E3 ubiquitin-protein ligase 1-like isoform X3 |
|  |  | TEA005473.1 | up | PREDICTED: uncharacterized protein LOC104609584 |
|  |  | TEA000833.1 | up | MYC2 trancription facor |
|  |  | TEA012504.1 | up | uncharacterized protein LOC110603959 isoform X1 |
|  |  | TEA019674.1 | up | aquaporin PIP2-1-like |
|  |  | TEA020517.1 | up | alpha carbonic anhydrase 1, chloroplastic |
|  |  | TEA025194.1 | up |  |
| MSTRG.152933.1 | up | TEA006218.1 | up | thioredoxin-like 3-1, chloroplastic |
|  |  | TEA008845.1 | up | probable plastidic glucose transporter 2 |
|  |  | TEA000833.1 | up | MYC2 trancription factor |
